# A Review of the Properties and Applications of Ozone in Endodontics: An Update

**Published:** 2013-05-01

**Authors:** Zahed Mohammadi, Sousan Shalavi, Mohammad Karim Soltani, Saeed Asgary

**Affiliations:** 1 Iranian Center for Endodontic Research, Research Institute of Dental Sciences, Shahid Beheshti University of Medical Sciences, Tehran, Iran; 2 Iranian National Elite Foundation, Tehran, Iran; 3 General Dental Practitioner, Hamedan, Iran; 4 Orthodontics Department, Hamedan University of Medical Sciences, Hamedan, Iran

**Keywords:** Antimicrobial, Dentin Bonding, Endodontics, Ozone, Toxicity

## Abstract

Ozone is a triatomic molecule consisting of three oxygen atoms. It is applied to oral tissues in the forms of ozonated water, ozonated olive oil and oxygen/ozone gas. This paper presents a brief review on the chemistry of ozone as well as its medical and dental applications focusing on its use in endodontics. Ozone’s antimicrobial activity, its effect on dentin bonding, toxicity and contra-indications are also reviewed.

## 1.Introduction

In 1839, Christian F. Schonbein, first noticed the emergence of a pungent gas with an electric smell. Later, in 1857, Wener Von Siemens designed an ozone generator. Oxygen/ozone therapy has a long history of research and clinical and therapeutic applications on humans. The first medical application was in 1870 when Lender purified blood in test tubes. Medical applications became widespread throughout Europe and America. As of 1929, more than 114 diseases were listed for treatment with oxygen/ozone. In 1930, Fisch, used ozone on a regular basis in his dental practice in Switzerland (1).

## 2.Chemistry of ozone

Ozone (O_3_) is a triatomic molecule with three oxygen atoms and molecular weight of 47.98 g/mol. Thermodynamically, this molecule is a highly instable compound that decomposes to pure oxygen with a short half-life in particular temperature and pressure conditions (2). Ozone is 1.6-fold denser and 10-fold more soluble in water (49 mL in 100 mL water at 0°C) than oxygen. Although ozone is not a radical molecule, it is the third most potent oxidant after fluorine and per sulfate. Ozone is an unstable gas that cannot be stored and should be used at once because it has a half-life of 40 min at 20°C (2). Ozone is naturally produced by the photo dissociation of molecular oxygen (O_2_) into activated oxygen atoms, which then react with further oxygen molecules. This transient radical anion rapidly becomes protonated, generating hydrogen trioxide (HO_3_), which, in turn, decomposes to an even more powerful oxidant, the hydroxyl radical (OH) (3). It is the fundamental form of oxygen that occurs naturally as a result of ultraviolet energy or lightning, causing a temporary recombination of oxygen atoms into groups of three. In the clinical setting, an oxygen/ozone generator simulates lightning via an electrical discharge field. Ozone gas has a high oxidation potential and is 1.5 times more effective than chloride when used as an antimicrobial agent against bacteria, viruses, fungi, and protozoa. It also has the capacity to stimulate blood circulation and the immune response. Ozone has been indicated for the treatment of 260 different pathologies (4, 5).

## 3.Routes of ozone therapy

Ozone is applied to oral tissues in the following forms: Ozonated water, ozonated olive oil, and oxygen/ozone gas. Ozonated water and olive oil have the capacity to entrap and then release oxygen/ozone; an ideal delivery system. These forms of application are used individually or in combination to treat dental disease (6).

## 4.Applications of ozone in medicine

Most of our knowledge is based on multiple case reports from hospitals and clinics. Ozone can enhance both lung function and inflammatory airway responses to inhale allergen in cases with pre-existing allergic airway diseases (7).

Medicated forms of O_3_ in a gaseous form are somewhat unusual, and that is why special application techniques have had to be developed for the safe use of ozone. In other words, due to the difficulty in handling and administering gaseous ozone, some methods and devices have been introduced to enhance its effectiveness. In local applications such as the treatment of external wounds, transcutaneous O_3_ gas bath has been established as a most practical and useful method; at low (subatmospheric) pressure in a closed system guaranteeing no escape of ozone into the ambient air. Ozonized water, whose use is particularly known in dental medicine, is optimally applied as a spray or in compressed form (8).

Apart from rectal insufflation which is principally used for the treatment of intestinal conditions, and also applied systemically, autohemotherapy has established itself as a systemic therapy of choice. A corresponding dosage of ozone gas is transferred (in the form of microbubbles) into 50 to 100 ml of the patient’s blood in a sealed, pressure-less system, thus achieving the finest possible distribution to reach the greatest possible number of red and white blood cells to activate their metabolism. This is a markedly low-risk method when hygiene guidelines are observed, disposable units are used, and the material used is ozone-resistant. In the therapy of pain in the locomotor system, ozone can be applied supportively in the form of intramuscular or intra-articular injections (2).

## 5.Ozone in dentistry

Muller et al. (9) compared the influence of ozone gas with photodynamic therapy (PDT) and known antiseptic agents (2% Chlorhexidine, 0.5 and 5% sodium hypochlorite solutions) on a multispecies oral biofilm in-vitro. The studied bacteria were *Actinomyces* *naeslundii*, *Veillonella* *dispar*, *Fusobacterium* *nucleatum, Streptococcus (S.) sobrinus, S. oralis and Candida (C.) albicans.* Gasiform ozone was produced by vacuum ozone delivery system Kavo HealOzone (Biberach, Germany). They concluded that the matrix-embedded microbial populations in biofilm are well protected against antimicrobial agents. Only 5% NaOCl solution was able to eliminate all bacteria effectively. Gasiform ozone or PDT was not able to significantly reduce or completely eliminate bacteria in the biofilm (9). Baysan et al. assessed antimicrobial effect of Kavo HealOzone device on primary root caries lesions (PRCL) and evaluated the efficiency of ozone specifically on *S. mutans and S. sobrinus* (10). As a result, ozone exposure to either 10 or 20 s under experimental conditions reduced the total levels of microorganisms in the PRCLs to <1% of the control values. Application of ozone for a period of 10 s was also capable of reducing the numbers of *S. mutans* and *S. sobrinus* in-vitro (10). Holmes (11) observed the effect of KaVo Healozone device on PRCL followed by professionally-applied remineralizing solution containing xylitol, fluoride, calcium, phosphate and zinc. This treatment modality was applied to 89 patients over 60. After 18 months 100% of ozone-treated primary root caries lesions (PRCLs) had improved. In control group, where lesions were left without treatment, only one PRCL had improved. In 62% of cases the status remained leathery, while in 37% of PCRL’s had worsened from leathery to soft texture (11).

The influence of ozonated water on the epithelial wound healing process in the oral cavity was observed by Filippi (12). It was found that ozonized water applied on a daily basis can accelerate the healing rate in oral mucosa. This effect was seen in the first two postoperative days. Daily treatment with ozonized water accelerates the physiological healing rate in the treated wounds as such changes were not seen in the untreated wounds (12).

## 6.Ozone in endodontics

Ozone gas in a ~4 g/m^3 ^concentration (HealOzone; KaVo, Biberach, Germany) is used clinically for endodontic treatments.

### 6.1.Antimicrobial activity

Most studies on the applications of ozone in endodontics focus on its antimicrobial activity. Nagayoshi et al. (13) found that ozonated water (0.5–4 mg/L) was highly effective in killing both gram positive and negative micro-organisms. Gram negative bacteria, such as *Porphyromonas (P.) endodontalis* and *P. gingivalis* were substantially more sensitive to ozonated water than gram positive oral streptococci and *C. albicans* in pure culture. Hems et al. (14) evaluated the potential of ozone as an antibacterial agent using *Enterococcus (E.) faecalis* as a test species. Ozone was used both as gasiform (produced by Pure zone device), and aqueous (optimal concentration 0.68 mg/L). They concluded that ozone in solution was antibacterial against planktonic *E. faecalis* after 240 s treatment. However it was not effective against E. faecalis within a biofilm unless they were displaced into the surrounding medium by agitation (14).

 Estrela et al. (15) studied antimicrobial effects of ozonated water, gaseous ozone and antiseptic agents (2.5% hypochlorite and 2% chlorhexidine) in infected human dental root canals. Over the 20 min contact time none of these substances had antibacterial effect against *E. faecalis* in the infected root canals.

Thanomsub et al. (16) tested the effects of ozone treatment on cell growth and ultrastructural changes in bacteria (*Escherichia coli, Salmonella sp., Staphylococcus aureus* and *Bacillus subtilis*). It was discovered that ozone at 0.167 mg/min/L concentration can be used to sterilize water, which is contaminated within 30 min with up to 105 cfu/ml bacteria. Destroying of bacterial cell membrane was observed, subsequently producing intercellular leakage and eventually causing cell lysis. Nevertheless, these ozone concentrations have no significant effect on the cell viability of bacterial cultures at higher concentrations of 106 and 107 CFU/mL (16).

 Polydorou et al. (17) found that 80 s as well as 40s applications of ozone was effective in eliminating *S. mutans*. In an animal study on the infected dog's teeth, Silveira et al. (2007) showed 77% root canal treatment success rate when using ozonized oil as an intra-canal medicament. A further study evaluated the effectiveness of ozonated water in the elimination of *C. albicans, E. faecalis,* and endotoxins from root canals (18). Findings revealed that ozonated water was effective against both *C. albicans* and *E. faecalis* immediately after treatment; however it did not have substantivity. Furthermore, ozonated water demonstrated no anti-endotoxin activity. The disinfecting effect of ozonized oxygen (120 s from the HealOzone) on E. faecalis has been assessed (19). Findings revealed that ozonized oxygen appears to be suitable for disinfecting root canal systems in cases where sodium hypochlorite is not indicated. Huth et al. (20) assessed the effectiveness of aqueous and gaseous ozone against *E. faecalis, C. albicans, Peptostreptococcus micros *and *Pseudomonas aeruginosa* cultured in planktonic media or in mono-species biofilms. Results demonstrated that high-concentrated gaseous and aqueous ozone was dose-, strain- and time-dependently effective against the tested microorganisms. The antibacterial activity of gaseous ozone was shown to be greater than KTP laser and less than NaOCl (21) and ozone gas delivered into irrigating fluids in the root canal may be useful as an adjunct for endodontic disinfection (22).

## 7.Effects on dentin bonding

Schmidlin et al. (23) evaluated the influence of direct high-dose gaseous ozone application (2100 ppm) on dentin and enamel shear bond strength; despite a possible retention of surface and subsurface oxide-related substances during high-dose ozone application, the strength was not impaired. Thus, adhesive restoration placement should be possible immediately after ozone application for cavity disinfection. However, other researchers reported that adhesion of the self-adhesive resin cement RelyX Unicem (3M ESPE, Seefeld, Germany) was significantly reduced after using gaseous ozone (24).

Magni et al. (25) indicated that Ozone gas did not compromise the mechanical properties of the adhesives [including Prime & Bond NT (Dentsply), Excite (Ivoclar-Vivadent), Syntac/Heliobond (Ivoclar-Vivadent) and Silorane System Adhesive (3 M-ESPE)]. When ozone gas was used to disinfect the cavity before a restoration, it had no influence on immediate enamel and dentin bond strength (26). Çehreli et al. (27) revealed that pre-treatment with ozone improved the marginal sealing ability of the fissure sealants; another study demonstrated that ozone therapy improved shear bond strength of AH-26 and EX Fill root canal sealers (28). Gurgan et al. (29) showed that ozone treatment did not impaired the shear bond strength of two self-etch adhesives (Clearfil SE Bond and Clearfil Tri-S Bond) used on coronal and radicular dentin. However other studies showed that ozone decreased the microtensile bond strength of dentin-composite resin interface (30) and reduced the initial microtensile bond strength of Clearfil SE Bond (31). According to Arslan et al. (32) ozone did not significantly affect the dentin bond strength of a silorane-based resin composite, filtek supreme. Also, another study revealed that ozone gas and ozonated water had no deleterious effects on the bond strengths and interfaces (33).

## 8.Toxicity

Ozone inhalation can be toxic to the pulmonary system and other organs. Complications caused by ozone therapy are infrequent at 0.0007 per application. Known side-effects are epiphora, upper respiratory irritation, rhinitis, cough, headache, occasional nausea, vomiting, shortness of breath, blood vessel swelling, poor circulation, heart problems and even stroke. Because of ozone's high oxidative power, all materials that come in contact with the gas must be ozone resistant, such as glass, silicon, and Teflon. However, in the event of ozone intoxication, the patient must be placed in the supine position and treated with vitamin E and N-acetylcysteine (2).

## 9.Contra-indications of ozone therapy

The following conditions mentioned in the medical literatures contraindicate ozone use e.g. acute alcohol intoxication, recent myocardial infarction, hemorrhage in any organ, pregnancy, hyperthyroidism, thrombocytopenia and ozone allergy (2-4).

## 10.Conclusion

Ozone is applied to oral tissues in various forms: ozonated water, ozonated olive oil, and oxygen/ozone gas. Ozone has been used in medicine extensively. In dentistry, its effectiveness on wound healing, antibacterial activity, and its effect on dentin bonding has been investigated. Ozone improves wound healing, assists in treating root caries and can be used against endodontic microbiota. Furthermore, it seems that ozone does not have significant adverse effect on dentin bonding. In spite of infrequency of side effects, ozone therapy may cause serious medical complications if incorrectly used. Therefore care must be taken when handling ozone.
